# Respiratory and Peripheral Muscle Weakness and Body Composition Abnormalities in Non-Cystic Fibrosis Bronchiectasis Patients: Gender Differences

**DOI:** 10.3390/biomedicines10020334

**Published:** 2022-01-31

**Authors:** Xuejie Wang, Ana Balaña-Corberó, Juana Martínez-Llorens, Liyun Qin, Yingchen Xia, Jianhua Zha, José María Maiques, Esther Barreiro

**Affiliations:** 1 Muscle Wasting and Cachexia in Chronic Respiratory Diseases and Lung Cancer Research Group, Pulmonology Department, Hospital del Mar-IMIM, Parc de Salut Mar, Parc de Recerca Biomèdica de Barcelona (PRBB), 08003 Barcelona, Spain; xuejie.wang@e-campus.uab.cat (X.W.); abalana@parcdesalutmar.cat (A.B.-C.); jmartinezl@psmar.cat (J.M.-L.); liyun.qin@e-campus.uab.cat (L.Q.); 361439919013@email.ncu.edu.cn (Y.X.); 361439918044@email.ncu.edu.cn (J.Z.); 2 Department of Medicine, Universitat Autònoma de Barcelona (UAB), 08035 Barcelona, Spain; 3 Health and Experimental Sciences Department (CEXS), Universitat Pompeu Fabra (UPF), 08002 Barcelona, Spain; 4 Centro de Investigación en Red de Enfermedades Respiratorias (CIBERES), Instituto de Salud Carlos III (ISCIII), 08003 Barcelona, Spain; 5 Department of Thoracic Surgery, The First Affiliated Hospital of Nanchang University, Nanchang 330006, China; 6 Radiology Department, Imatge Mèdica Intercentres, Parc de Salut Mar, Hospital del Mar, 08003 Barcelona, Spain; jmaiques@psmar.cat

**Keywords:** bronchiectasis patients, upper limb muscle function, lower limb muscle function, respiratory muscle function, muscle weakness, differences between male and female patients, radiological extension

## Abstract

As demonstrated in COPD, bronchiectasis patients may experience respiratory and peripheral muscle dysfunction. We hypothesized that respiratory and peripheral (upper and lower limbs) muscle function and nutritional status may be more significantly altered in female than in males for identical age and disease severity. In mild-to-moderate bronchiectasis patients (*n* = 150, 114 females) and 37 controls (*n* = 37, 21 females), radiological extension, maximal inspiratory and expiratory pressures (MIP and MEP), sniff nasal inspiratory pressure (SNIP), hand grip and quadriceps muscle strengths, body composition, and blood analytical biomarkers were explored. Compared to the controls, in all bronchiectasis patients (males and females), BMI, fat-free mass index (FFMI), fat tissue, upper and lower limb muscle strength, and respiratory muscle strength significantly declined, and FFMI, fat tissue, and quadriceps muscle function were significantly lower in female than male patients. In patients with mild-to-moderate bronchiectasis, respiratory and peripheral muscle function is significantly impaired and only partly related to lung disease status. Quadriceps muscle strength was particularly weakened in the female patients and was negatively associated with their exercise tolerance. Muscle weakness should be therapeutically targeted in bronchiectasis patients. Body composition and peripheral muscle function determination should be part of the comprehensive clinical assessment of these patients.

## 1. Introduction

Non-cystic fibrosis (CF) bronchiectasis is a chronic respiratory condition characterized by abnormalities of the airways that facilitate the collection of lung secretions in the patients, which further deteriorates the underlying structure. Remodeling of the airways is frequent in these patients, along with other events, such as chronic inflammation and bacterial colonization [1,2]. The prevalence of bronchiectasis is progressively increasing as more diagnostic tools become available [3,4].

Extrapulmonary manifestations are common among patients with chronic respiratory diseases, including those with bronchiectasis [5,6,7,8]. The analysis of skeletal muscle dysfunction and sarcopenia of patients with chronic obstructive pulmonary disease (COPD) have been a matter of research in multiple previous investigations [9,10,11,12,13,14]. Peripheral muscle weakness, particularly of the lower limbs, takes place in up to one third of COPD patients, even in those with a mild airway obstruction [10,15,16,17,18]. Respiratory muscle dysfunction is also common in COPD patients [19,20,21,22]. Several clinical factors are involved in the pathophysiology of skeletal muscle dysfunction and sarcopenia in COPD [9,10,11,12,13,14]. Inactivity, deconditioning, and nutritional abnormalities, including vitamin D deficiency, are counted among the most relevant contributors to muscle dysfunction and atrophy in these patients [9,10,11,12,13,14,23]. For instance, acute exacerbations, which are frequent in COPD and bronchiectasis patients, negatively impact on their quality of life and disease prognosis as a result of reduced physical and muscle activity [9,10,11,12,13,14]. Whether the function of respiratory and limb muscles may be altered in patients with mild-to-moderate bronchiectasis remains to be thoroughly understood.

The study of the potential differences between female and male patients in chronic respiratory disease has gained great attention in biomedical research in the last decade [6]. As such, it has been recently demonstrated that in a large-cohort of bronchiectasis patients, females exhibited a less severe disease along with a better profile of inflammatory biomarkers than the male patients recruited in the same investigation [6]. Disease outcomes may also differ between male and female patients in chronic respiratory patients [24]. Several factors may account for the reported differences observed between female and male chronic respiratory patients. Lung and airway anatomy, chronic infection and inflammation, differences in host defense mechanisms, and environmental factors, including physical activity and nutritional abnormalities, are a few factors that contribute to the gender differences observed in bronchiectasis patients [6,25,26]. In patients with advanced COPD, peripheral muscle dysfunction and damage was significantly more prominent among female patients compared to men [27]. Whether similar findings can be observed in female patients with bronchiectasis remains to be answered.

On this basis, we hypothesized that respiratory and peripheral muscle function, (both upper and lower limbs) and nutritional status (body compartments) may be more significantly altered in female patients than in males for the same age and disease severity. As such, the following objectives were established. In female and male patients with mild-to-moderate bronchiectasis compared to a group of healthy control subjects, parameters assessing respiratory and peripheral muscle function were determined: (1) maximal inspiratory and expiratory pressures (MIP and MEP, respectively), (2) sniff nasal inspiratory pressure (SNIP), (3) hand grip and quadriceps muscle strengths, (4) body composition, (5) blood analytical biomarkers, and (6) correlations between lung function and the extrapulmonary parameters.

## 2. Methods

### 2.1. Study Population

Patients with stable non-CF bronchiectasis (*n* = 150, 114 female) were consecutively recruited from the Bronchiectasis Multidisciplinary Unit at the Hospital del Mar (Barcelona, Spain). Moreover, a group of non-smoker healthy age-matched control subjects (*n* = 37, 21 females) was also recruited from the general population (patients’ relatives). All the patients had a primary diagnosis of bronchiectasis on the basis of high-resolution computerized tomography (HRCT) and published guidelines were followed [4,28,29]. Patients did not have any acute exacerbation at least three months prior to study entry. Habitual medication taken by the patients was maintained throughout the duration of the study: inhaled bronchodilators with and without inhaled corticosteroids, inhaled antibiotics in a few cases, and mucolytics. Patients were consecutively recruited from the Bronchiectasis Clinic and followed a regular Mediterranean diet, as it is common in this geographical area. Patients were sedentary and were not following any specific exercise training program or going to the gymnasium at the time of study entry. Likewise, healthy control subjects were also sedentary and were not practicing any high-intensity outdoor or indoor exercise program, and they were also following a regular Mediterranean diet.

Exclusion criteria were as follows: acute or chronic respiratory failure [30], COPD [31], other chronic respiratory diseases, including asthma, coronary heart disease, limiting osteoarticular condition, chronic metabolic diseases of any etiology, presence of paraneoplastic syndrome, myopathies, treatment with oral steroids, or other drugs that had potential effects on muscle structure or function.

Nutritional status, lung function, respiratory and peripheral muscle functions, exercise capacity, and blood parameters were determined in both bronchiectasis patients and the control subjects. This was a prospective, cross-sectional study in which patients were recruited for two years (July 2019–June 2021).

### 2.2. Ethics

The current study was designed following the guidelines of the World Medical Association for Research in Humans (Seventh revision of the Declaration of Helsinki, Fortaleza, Brazil, 2013) [32] and the ethical standards on human experimentation in our institution. The study was approved by the Institutional Ethics Committee on Human Investigation before the start. (Hospital del Mar-IMIM, Barcelona, project number 2019/8955/I). An informed written consent was obtained from both patients and control subjects. Finally, the participation of all participants was confidential and voluntary at all times.

### 2.3. Bronchiectasis Severity Scores

The FACED (FEV_1_, age, chronic colonization, extension, dyspnea), EFACED (exacerbation FACED), and BSI (bronchiectasis severity index) scores were used to assess the disease severity of bronchiectasis patients [33,34,35].

### 2.4. Radiological Extension of Bronchiectasis

The radiological extension of bronchiectasis was evaluated by means of HRCT-scans in all the study patients. Scores for each patient were calculated by two independent observers according to previously established criteria [7,36,37]. The extent of bronchiectasis (ES) was scored for each lobe as follows: grade 0 = no disease; grade 1 = one or partial bronchopulmonary segment involved; grade 2 = two or more bronchopulmonary segments involved. The lingula lobe was considered as an independent one in this analysis. The bronchial dilatation (DS) was quantified relative to the adjacent pulmonary arteries as follows: grade 0 = no bronchiectasis; grade 1 = less than twice (200%) diameter of adjacent pulmonary artery (APA); grade 2 = 200–300% diameter of APA; grade 3 ≥ 300% diameter of APA. Bronchial wall thickness (TS) was scored as follows: grade 0 = none; grade 1 = 50% of APA, grade 2 = 50–100% of APA; grade 3 ≥ 100% of APA.

The CT scans were also scored for parenchymal items in the six lobes: collapse or consolidation, mucus plugging, emphysema, and fibrosis or retraction. Collapse or consolidation: subsegmental collapse or consolidations = 1 and segmental or lobar collapse or consolidations = 2. Mucus plugging was scored as follows: the presence of subsegmental mucus plugging = 1 and in case of the presence of segmental or lobar mucus plugging = 2. Emphysema was recorded as follows: subsegmental emphysema = 1 and segmental or lobar emphysema = 2. Fibrosis or retraction was scored as follows: subsegmental fibrosis or retraction = 1 and segmental, lobar fibrosis, or retraction = 2.

Global scores of both lungs were taken for extension, bronchial dilatation, and bronchial wall thickness. The total extent of bronchiectasis (TES) was taken as the sum of the ES for each of the six lobes. The global severity of bronchial dilatation (GDS) was estimated using a weighted average, calculated as the “sum of the extent score multiplied by the dilatation score for each lobe”, divided by the “total extent score” (GDS = ∑(ES × DS)_1–6_/TES). Similarly, the global severity of bronchial wall thickness (GWTS) was estimated as the “sum of the extent score multiplied by the thickness score for each lobe” divided by the “total extent score” (GTS = ∑(ES × TS)_1–6_/TES).

### 2.5. Nutritional and Body Assessment

Body weight and height were measured after a fasting period of at least four hours in all the patients and healthy controls. Nutritional evaluation included body mass index (BMI), determination of the fat-free mass index (FFMI) using bioelectrical impedance (Bodystat 1500, Bodystat Ltd., Isle of Man, British Isles), and conventional blood markers [12,13]. The following outcomes were measured using bioelectrical impedance: fat-free mass (FFM), FFM index (FFMI), and fat tissue absolute and percentage values.

#### 2.5.1. Lung Function Assessment

Lung function was evaluated through determination of prebronchodilator spirometric values (COVID-19 pandemic period), static lung volumes, and diffusion capacity using standard procedures, equipment, and established reference values [38,39,40,41].

#### 2.5.2. Limb Muscle Function

Upper limb muscles—Handgrip strength was evaluated using a specific dynamometer (Jamar 030J1, Chicago, IL, USA). The maximum voluntary contraction of the flexor muscles of the non-dominant hand was assessed. The highest value out of three reproducible maneuvers (<5% variability among them) was accepted as the valid measurement for each subject [42,43]. Reference values from Luna-Heredia et al. [42] were used in the analysis.

Lower limb muscles—In both patients and controls, quadriceps muscle strength was evaluated through the determination of isometric maximum voluntary contraction (QMVC) of the non-dominant lower limb, as formerly described [12,13]. Briefly, an isometric dynamometer (Biopac Systems, Goleta, CA, USA) connected to a digital polygraph (Biopac Systems) was used for these measurements. Individuals had to lie on their back on a stretcher, while the non-dominant ankle was fixed with a strap. Subjects had their lower limbs falling down at 90° from the stretcher. The ankle attached to the strap performed the maneuver to calculate quadriceps muscle strength. The highest value out of three brief reproducible maneuvers (<5% variability among them) was accepted as the QMVC for each subject. Reference values from Seymour et al. [15] were used in the analysis.

### 2.6. Respiratory Muscle Evaluation

Maximal inspiratory pressure (MIP)—MIP at the mouth was performed from the residual volume (RV). The measurements were taken when participants were in a sitting position. In order to measure the MIP, an occludable oral piece with a small orifice was used to minimize the participation of the buccinator muscles [44]. The oral piece was connected to a pressure manometer (TSD 104, Biopac Systems), whose signal was registered by a digital polygraph (Biopac Systems). The MIP final outcome for each subject was obtained from the highest value out of three reproducible maneuvers (a difference <5% among them), as also previously described [44,45]. Reference values from Araújo et al. [45] were used in the analysis.

Sniff nasal inspiratory pressure (SNIP)—SNIP was also recorded using a pressure transducer connected to a catheter placed in the nostril during the measurement of a SNIP maneuver [45,46]. The subject was instructed to sniff quickly and deeply. The SNIP final outcome for each subject was obtained from the highest value out of ten reproducible maneuvers (a difference < 5% among them), as also previously described [44,45]. Reference values from Araújo et al. [45] were used in the analysis.

Maximal expiratory pressure (MEP)—MEP at the mouth was measured from total lung capacity (TLC). In order to measure the MEP, an occludable oral piece with a small orifice, used to minimize the participation of the buccinator muscles, was connected to a pressure manometer (TSD 104, Biopac Systems), whose signal was recorded by a digital polygraph (Biopac Systems). All the measurements were performed in a sitting position. The MEP final outcome for each subject was obtained from the highest value out of three reproducible maneuvers (a difference <5% among them), as also previously described [44,45]. Reference values from Araújo et al. [45] were used in the analysis.

#### Exercise Capacity

Exercise capacity was assessed through the measurement of the six-minute walking distance following current guidelines [47,48,49]. Encouragement was given to all the subjects during the test. Reference values from Enright et al. [49] were used in the analysis.

### 2.7. Statistical Analysis

The normality of the distribution of the study variables was assessed using the Shapiro–Wilk test. A minimum number of 60 patients and 12 healthy control subjects was required to achieve an 80% statistical power for the target variable (FFMI) assuming a standard deviation of 2.2 and alpha risk of 0.05. The study variables are presented as mean (standard deviation) in figures and tables. Both control subjects and patients were subdivided according to genders (21 and 114 females, control subjects and patients, respectively). The differences between the two groups were assessed using the Student’s *t*-test or Mann–Whitney U test. Differences between the control and bronchiectasis patients within each gender group and between gender (only patients) were explored using one-way analysis of variance (ANOVA) or Kruskal–Wallis tests and Tukey’s post hoc to correct for multiple comparisons. The Chi-square test was used for the categorical variables (smoking history). In summary, the following comparisons were assessed: (1) bronchiectasis patients and healthy controls as a whole, (2) differences between female and male patients in bronchiectasis patients, (3) differences between female controls and female patients, and (4) differences between male controls and male patients. Potential differences between female and male controls were not assessed in this study, as this was not part of the study hypothesis or objectives. The statistical significance was established as *p* < 0.05. Statistical analysis was performed using the software SPSS version 23 (SPSS Inc, Chicago, IL, USA). Correlations are shown in graphical correlation matrixes for all the patients, obtained from the R package corrplot (https://cran.r-project.org/web/packages/corrplot/index.html, accessed on 15 October 2021), in different colors: blue for positive correlations and red for negative ones.

## 3. Results

### 3.1. Clinical Characteristics of the Study Subjects

Table 1 and Table 2 illustrate the clinical characteristics of the study population. Age did not differ between patients and control subjects. Healthy controls were non-smokers. BSI and EFACED scores were lower among the male patients compared to female patients (Table 2). Very few patients (12.7%) had chronic colonization by Pseudomonas aeruginosa (PA) and no differences were observed between male and female patients (Table 1 and Table 2). Radiological extension of bronchiectasis was greater in male than in female patients (Table 1 and Table 2).

**Figure 1:**
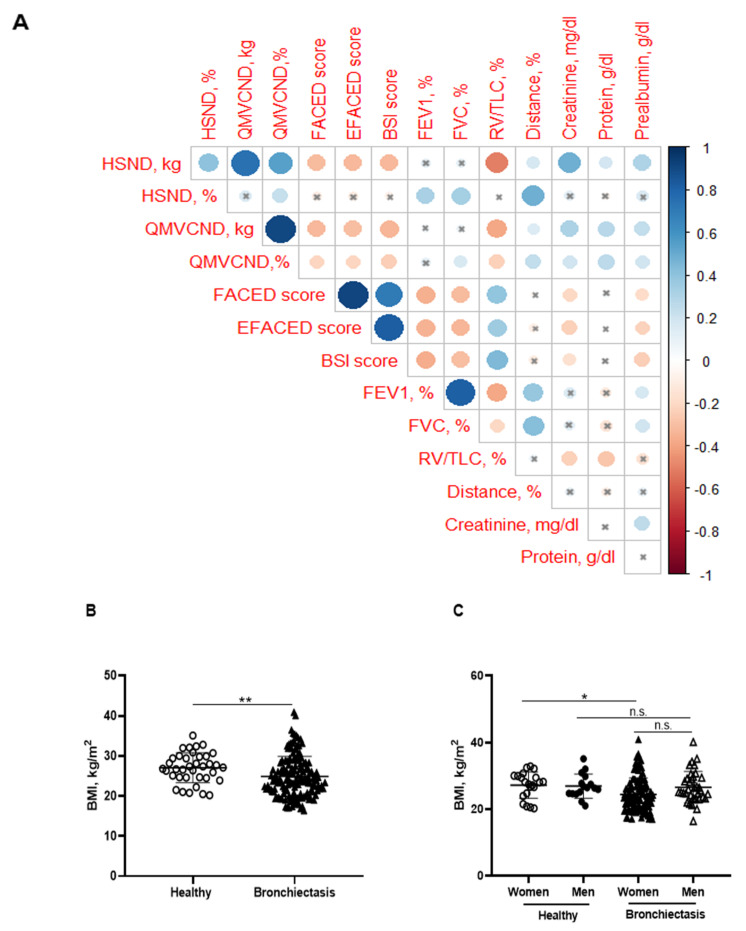
(**A**) Correlation matrix of clinical variables and peripheral muscle strength variables, in which positive correlations are represented in blue, while negative correlations are represented in red. The intersection within the circle represents a *p* value > 0.05. Color intensity and the size of the circle are proportional to the correlation coefficients, as indicated in the Y axis on the right-hand side of the graph. (**B**) Mean values and standard deviation of body mass index (BMI) (kg/m^2^) in healthy controls and bronchiectasis patients. (**C**) Mean values and standard deviation of BMI (kg/m^2^) in both female and male healthy controls and bronchiectasis patients. Statistical analyses and significance: ** *p* ≤ 0.01 between bronchiectasis patients and healthy controls. * *p* ≤ 0.05 between healthy and bronchiectasis women and n.s., non-significant differences between healthy and bronchiectasis men or between men and women patients.

Significant positive correlations were detected between FACED, EFACED, and BSI with RV/TLC among all the patients (Figure 1A). Patients as a whole and when divided by gender exhibited mild-to-moderate airway obstruction, reduced diffusion capacity, airway trapping, and decreased exercise capacity compared to the healthy controls (Table 1 and Table 2). Additionally, female patients had a greater degree of airway trapping as determined by RV and RV/TLC and worse exercise capacity (distance absolute values) than male patients (Table 2). In the blood compartment, a significant rise in acute phase reactants (CRP, ESR, fibrinogen, and alpha-1 antitrypsin) was observed in the patients compared to control subjects (Table 1). No significant differences in levels of these parameters were observed between male and female patients in this cohort (Table 2). Moreover, levels of hemoglobin, albumin, and prealbumin were mildly reduced in the patients as a whole compared to the controls (Table 1). In the male patients, levels of these parameters were significantly greater than those detected in the female patients (Table 2). Furthermore, significant negative associations were observed between FACED, EFACED, and BSI scores, and creatinine and prealbumin blood parameters (Figure 1A). No significant differences in levels of inflammatory or nutritional parameters were detected when patients were divided according to chronic colonization by PA (data not shown).

### 3.2. Body Composition

**Figure 2:**
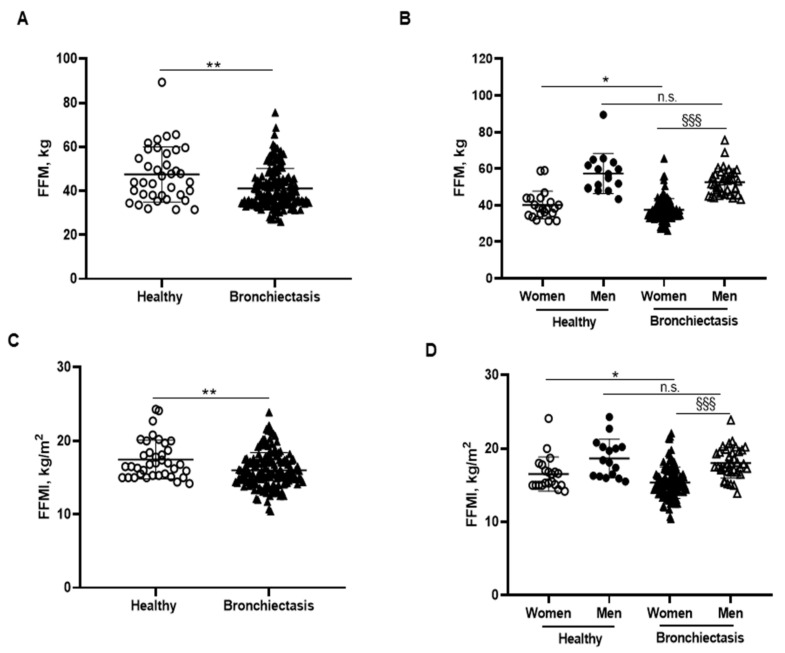
(**A**) Mean values and standard deviation of FFM (kg) in healthy controls and bronchiectasis patients. (**B**) Mean values and standard deviation of FFM (kg) in both female and male healthy controls and bronchiectasis patients. (**C**) Mean values and standard deviation of FFMI (kg/m^2^) in healthy controls and bronchiectasis patients. (**D**) Mean values and standard deviation of FFMI (kg/m^2^) in both female and male healthy controls and bronchiectasis patients. Statistical analyses and significance: ** *p* < 0.01 between bronchiectasis patients and healthy controls. * *p* ≤ 0.05 between healthy and bronchiectasis women; n.s., non-significant differences between healthy and bronchiectasis men; and §§§ *p* ≤ 0.001 between men and women patients.

**Figure 3:**
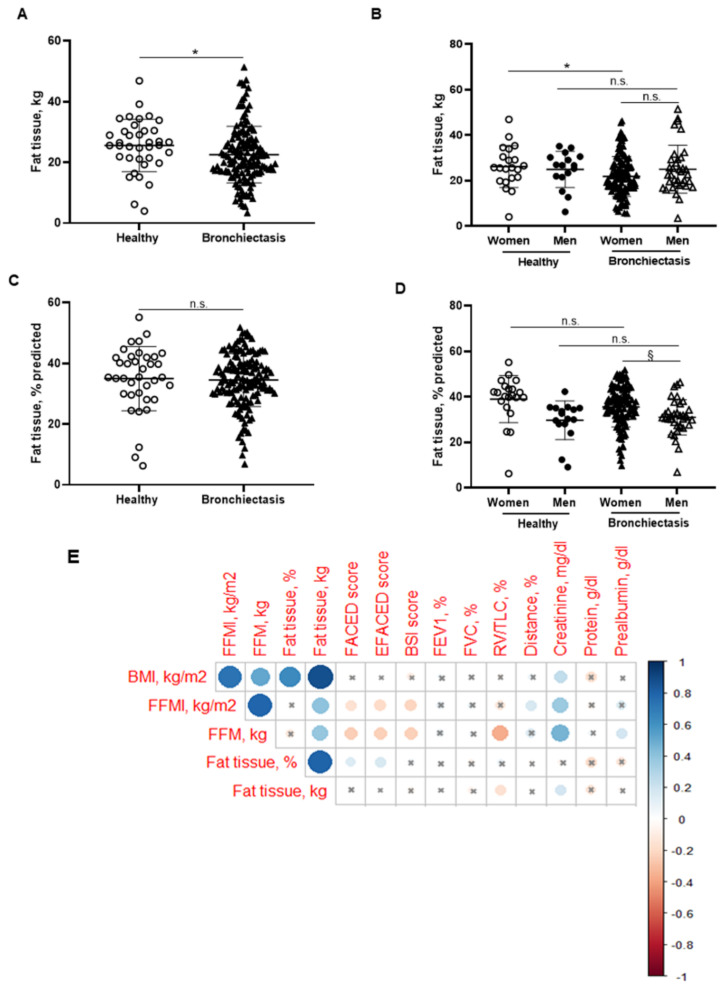
(**A**) Mean values and standard deviation of fat tissue (kg) in healthy controls and bronchiectasis patients. (**B**) Mean values and standard deviation of fat tissue (kg) in both female and male healthy controls and bronchiectasis patients. (**C**) Mean values and standard deviation of fat tissue (% predicted) in healthy controls and bronchiectasis patients. (**D**) Mean values and standard deviation of fat tissue (% predicted) in both female and male healthy controls and bronchiectasis patients. (**E**) Correlation matrix of clinical variables and body composition variables, in which positive correlations are represented in blue, while negative correlations are represented in red. The intersection within the circle represents *p* value > 0.05. Color intensity and the size of the circle are proportional to the correlation coefficients, as indicated in the Y-axis on the right-hand side of the graph. Statistical analyses and significance: * *p* ≤ 0.05 and n.s. (non-significant differences) between bronchiectasis patients and healthy controls. * *p* ≤ 0.05 and n.s. between healthy and bronchiectasis women or between healthy and bronchiectasis men; and § *p* ≤ 0.05 and n.s. between men and women patients.

BMI was significantly reduced in the patients compared to the healthy subjects, particularly in the female patients (Figure 1B,C, respectively). No significant differences were observed between male patients and healthy male controls (Figure 1C). A significant reduction in FFM was observed in all the patients compared to healthy controls (Figure 2A). Moreover, FFM was lower in female patients than in healthy females, while no differences were observed in the males (Figure 2B). FFM was greater in the male than in the female patients (Figure 2B). FFMI significantly decreased in the patients as a whole compared to the healthy controls (Figure 2C). Importantly, in female patients, FFMI was also significantly reduced compared to female controls (Figure 2D). Moreover, male patients exhibited a greater FFMI than female patients (Figure 2D). Fat tissue was significantly lower in patients as a whole and in the female patients than in the healthy controls (Figure 3A,B, respectively). The percentage of fat tissue was significantly lower in the male than in the female patients, while no differences were observed in the other groups (Figure 3C,D). In the overall study patients, BMI positively correlated with FFMI, FFM, absolute and percentage fat tissue, and blood creatinine (Figure 3E). Among the study patients, FFM and FFMI parameters were inversely correlated with FACED, EFACED, and BSI, whereas they were positively associated with creatinine blood levels (Figure 3E). No significant differences in the body composition parameters were detected when patients were divided according to chronic colonization by PA (data not shown).

### 3.3. Upper Limb Muscle Strength

**Figure 4:**
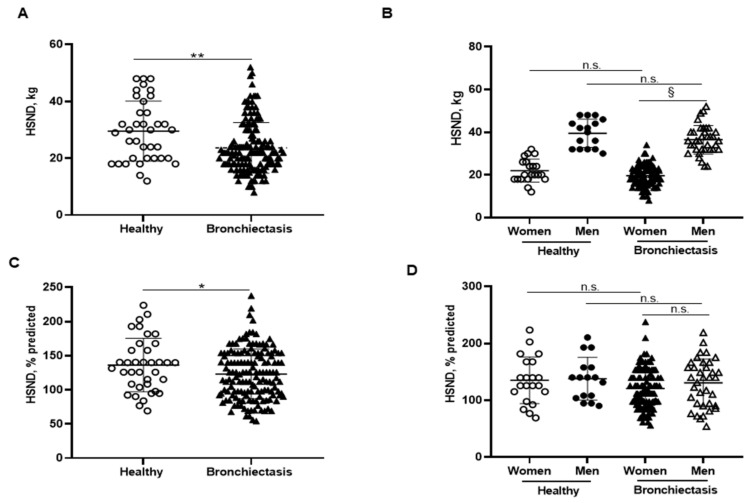
(**A**) Mean values and standard deviation of handgrip strength non-dominant (HSND) (kg) in healthy controls and bronchiectasis patients. (**B**) Mean values and standard deviation of HSND (kg) in both female and male healthy controls and bronchiectasis patients. (**C**) Mean values and standard deviation of HSND (% predicted) in healthy controls and bronchiectasis patients. (**D**) Mean values and standard deviation of HSND (% predicted) in both female and male healthy controls and bronchiectasis patients. Statistical analyses and significance: * *p* ≤ 0.05 and ** *p* ≤ 0.01 between bronchiectasis patients and healthy controls. n.s., non-significant differences between healthy and bronchiectasis women or between healthy and bronchiectasis men; § *p* ≤ 0.05 and n.s. between men and women patients.

Handgrip absolute strength values were significantly reduced in the patients as a whole compared to the healthy controls, particularly among the female patients compared to male patients (Figure 4A,B, respectively). Predicted handgrip strength values were also significantly lower in the patients as a whole than in the controls, while no differences were observed when patients were subdivided according to gender (Figure 4C,D, respectively). Interestingly, handgrip absolute values negatively correlated with FACED, EFACED, and BSI scores and RV/TLC, while it positively correlated with exercise capacity (distance walked), creatinine, protein, and prealbumin blood levels among the overall patients (Figure 1A).

### 3.4. Lower Limb Muscle Strength

**Figure 5:**
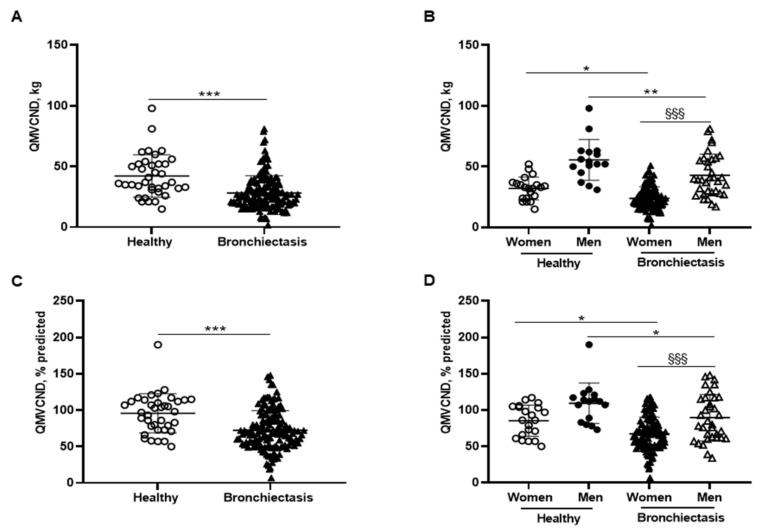
(**A**) Mean values and standard deviation of quadriceps maximal strength during maximum voluntary contraction in the non-dominant leg (QMVC) (kg) in healthy controls and bronchiectasis patients. (**B**) Mean values and standard deviation of QMVC (kg) in both female and male healthy controls and bronchiectasis patients. (**C**) Mean values and standard deviation of QMVC (% predicted) in healthy controls and bronchiectasis patients. (**D**) Mean values and standard deviation of QMVC (% predicted) in both female and male healthy controls and bronchiectasis patients. Statistical analyses and significance: *** *p* ≤ 0.001 between bronchiectasis patients and healthy controls. * *p* ≤ 0.05; ** *p* ≤ 0.01 between healthy and bronchiectasis women or between healthy and bronchiectasis men; §§§ *p* ≤ 0.001 between men and women patients.

Patients as a whole exhibited a significant decline in absolute and predicted values of QMVC compared to healthy controls, which was also confirmed when patients were subdivided into male and female genders (Figure 5A–D, respectively). Additionally, QMVC absolute and predicted values were significantly lower in the female compared to the male patients (Figure 5B,D, respectively). QMVC absolute and predicted values negatively correlated with FACED, EFACED, and BSI scores and RV/TLC, while they positively correlated with exercise capacity (distance walked), creatinine, protein, and prealbumin blood levels among the overall patients (Figure 1A).

### 3.5. Inspiratory and Expiratory Muscle Strength

**Figure 6:**
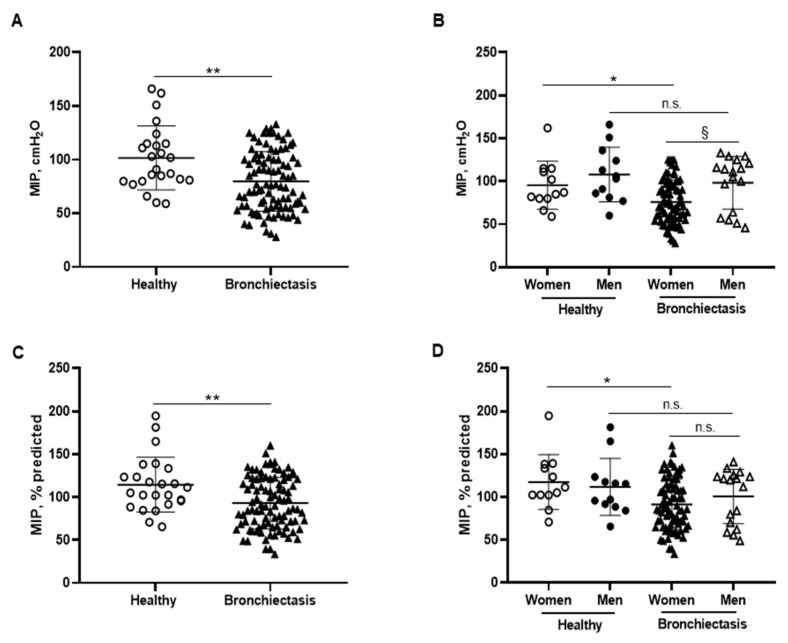

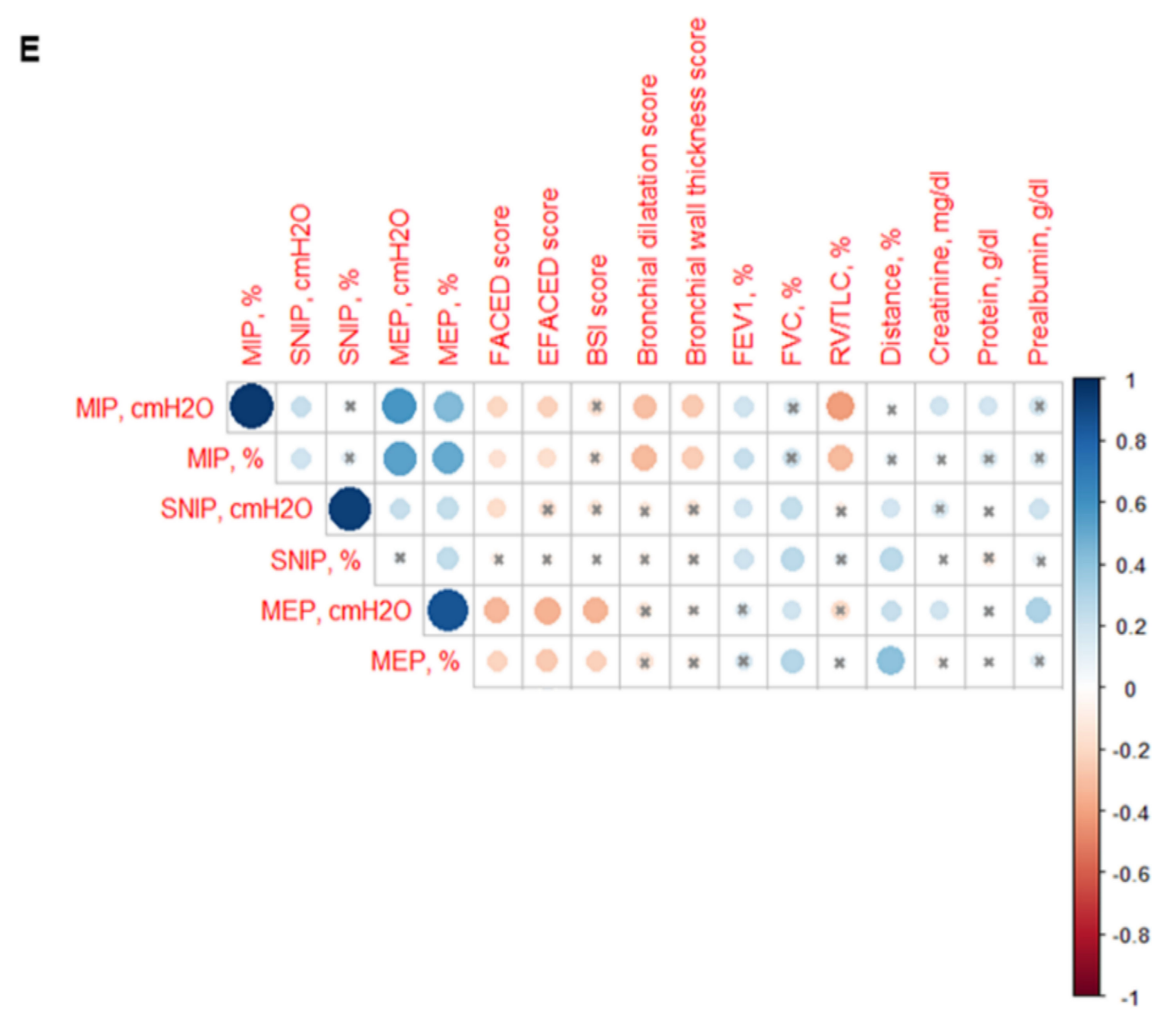
(**A**) Mean values and standard deviation of maximal inspiratory pressure (MIP) (cmH_2_O) in healthy controls and bronchiectasis patients. (**B**) Mean values and standard deviation of MIP (cmH_2_O) in both female and male healthy controls and bronchiectasis patients. (**C**) Mean values and standard deviation of MIP (% predicted) in healthy controls and bronchiectasis patients. (**D**) Mean values and standard deviation of MIP (% predicted) in both female and male healthy controls and bronchiectasis patients. (**E**) Correlation matrix of clinical variables and respiratory muscle strength variables, in which positive correlations are represented in blue, while negative correlations are represented in red. The intersection within the circle represents *p* value > 0.05. Color intensity and the size of the circle are proportional to the correlation coefficients, as indicated in the Y-axis on the right-hand side of the graph. Statistical analyses and significance: ** *p* ≤ 0.01 between bronchiectasis patients and healthy controls. * *p* ≤ 0.05 between healthy and bronchiectasis women and n.s., non-significant differences between healthy and bronchiectasis men; § *p* ≤ 0.05 and n.s. between men and women patients.

**Figure 7:**
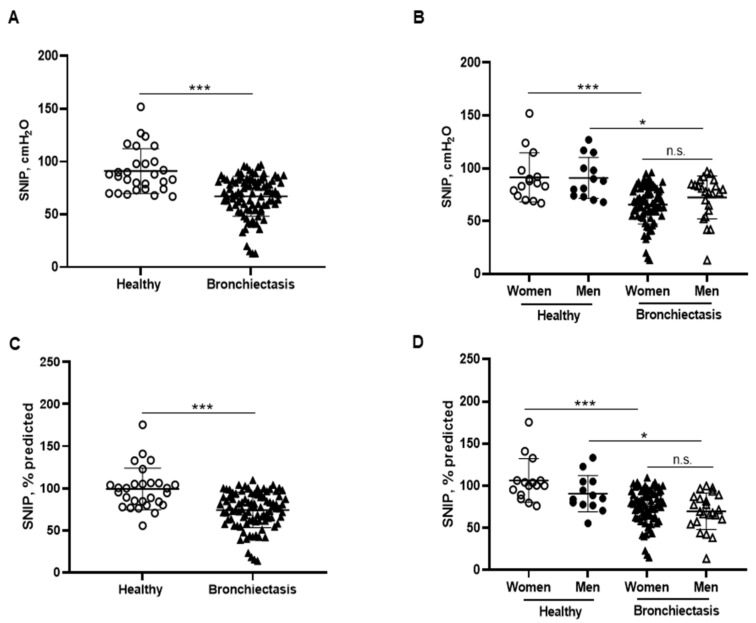
(**A**) Mean values and standard deviation of maximal sniff nasal pressure (SNIP) (cmH_2_O) in healthy controls and bronchiectasis patients. (**B**) Mean values and standard deviation of SNIP (cmH_2_O) in both female and male healthy controls and bronchiectasis patients. (**C**) Mean values and standard deviation of SNIP (% predicted) in healthy controls and bronchiectasis patients. (**D**) Mean values and standard deviation of SNIP (% predicted) in both female and male healthy controls and bronchiectasis patients. Statistical analyses and significance: *** *p* ≤ 0.001 between bronchiectasis patients and healthy controls. *** *p* ≤ 0.001 between healthy and bronchiectasis women; * *p* ≤ 0.05 between healthy and bronchiectasis men; n.s., non-significant differences between men and women patients.

MIP absolute and predicted values significantly decreased in the patients as a whole compared to the healthy controls and this was also confirmed in the female patients compared to the female controls (Figure 6A–D). In addition, MIP absolute values were significantly greater in the male than in the female patients (Figure 6B). Among the study patients, negative associations were detected between MIP absolute and predicted values and FACED, EFACED, RV/TLC, bronchial dilatation, and wall thickness scores (Figure 6E). SNIP absolute and predicted values were significantly diminished in the patients as a whole and in both male and female patients compared to the respective healthy controls (Figure 7A–D).

**Figure 8:**
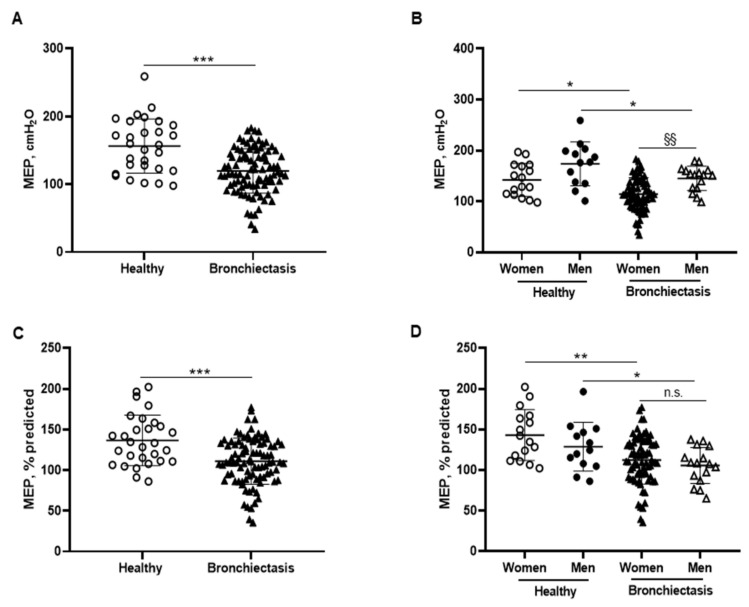
(**A**) Mean values and standard deviation of maximal expiratory pressure (MEP) (cmH_2_O) in healthy controls and bronchiectasis patients. (**B**) Mean values and standard deviation of MEP (cmH_2_O) in both female and male healthy controls and bronchiectasis patients. (**C**) Mean values and standard deviation of MEP (% predicted) in healthy controls and bronchiectasis patients. (**D**) Mean values and standard deviation of MEP (% predicted) in both female and male healthy controls and bronchiectasis patients. Statistical analyses and significance: *** *p* ≤ 0.001 between bronchiectasis patients and healthy controls. * *p* ≤ 0.05; ** *p* ≤ 0.01 between healthy and bronchiectasis women or between healthy and bronchiectasis men; §§ *p* ≤ 0.01 and n.s., non-significant differences between men and women patients.

Absolute and predicted MEP values were significantly lower in the patients as a whole and in both male and female patients than in the respective control subjects (Figure 8A–D). Furthermore, MIP absolute values were significantly increased in the male compared to the female patients (Figure 8B). In the overall study patients, MEP absolute and predicted values were negatively associated with FACED, EFACED, and BSI scores, while they positively correlated with FVC and exercise capacity (distance walked, Figure 6E).

## 4. Discussion

Patients with mild-to-moderate bronchiectasis exhibited airflow limitation, a decrease in diffusion capacity along with air trapping, reduced exercise capacity, and a slight increase in blood inflammatory parameters as also previously demonstrated [6,7]. The most relevant findings in the study are discussed below.

Despite body composition parameters being within the normal range (BMI, 24.9 kg/m^2^ and FFMI, 16 kg/m^2^) among the study patients, they were significantly reduced when compared to a population of healthy subjects (37 recruited for the purpose of the investigation). Importantly, FFM along with BMI and FFMI, were also significantly lower in the patients than in the healthy controls. Importantly, the female patients were those exhibiting the greater reduction in those parameters compared to the healthy female controls. Interestingly, male patients exhibited a significant increase in FFM and FFMI values compared to the female patients. These findings suggest that gender differences exist with regards to the levels of FFM between male and female patients for the same degree of disease severity. Similarly, gender differences have also been reported in several nutritional and inflammatory parameters as well as in BMI between female and male bronchiectasis patients [6,50].

Bronchiectasis patients in this large cohort demonstrated significant inverse associations between body composition parameters (FFM and FFMI), nutritional biomarkers (creatinine and prealbumin), and disease severity as measured by FACED, EFACED, and BSI scores. However, no relationships were observed between nutritional status and lung function parameters among the patients. These results are consistent with previous findings in which no significant associations were demonstrated between lung function and nutritional parameters [51]. Moreover, no differences were observed in body composition or nutritional parameters between patients with chronic colonization by PA (12.7%) and those without, suggesting that this factor did not influence those results in this cohort of patients (data not shown). Importantly, a decline in fat tissue content was observed in all the patients as a whole and particularly among the female patients when the parameter was expressed in kg. No differences were observed between male patients and healthy male subjects in these parameters.

A recent investigation [52] has demonstrated that sedentary behavior and a low number of daily steps predicted the risk of hospitalizations in one year among patients with bronchiectasis compared to patients with a more active lifestyle. In the present study, the distance walked among bronchiectasis patients, both male and females, was also reduced compared to the healthy controls. In addition, total distance walked by female patients was significantly lower than that observed in the male patients, as also previously shown [53]. These results imply that exercise tolerance is an important parameter that should be included in the management of patients with bronchiectasis in clinics.

Skeletal muscle dysfunction is a major systemic manifestation in patients with chronic diseases, including COPD, chronic heart and kidney failure, and cancer [9,10,11,12,13,14]. Specifically, one third of COPD patients exhibit muscle weakness of the lower limbs irrespective of the degree of the airway obstruction [15]. Despite that COPD patients may present upper and lower limb muscle dysfunction, the latter are most commonly evaluated in clinical settings due to its implications in their exercise tolerance [9,10,11,12,13,14,54]. Muscle contractile performance in vivo can be identified through the assessment of either muscle strength or resistance, and the former is most widely evaluated in clinics [9,10,11,12,13,14]. In the current investigation, measurements of upper muscle function demonstrated a significant decline in handgrip strength in the bronchiectasis patients compared to the healthy controls. Strength of the upper limb muscles did not show, however, any significant gender differences.

Muscle weakness of the quadriceps was observed in the bronchiectasis patients compared to the controls. Importantly, a significant decrease in quadriceps muscle strength, absolute and predicted values, was observed in both female and male patients compared to their respective healthy control subjects. Weakness of the upper and lower limb muscles was also observed in bronchiectasis patients of a younger age and severe airway obstruction [55]. Moreover, in the present study, significant positive correlations were observed between quadriceps muscle function (absolute and predicted) and the predicted distance walked in the six-minute walk test. It is also worth mentioning that despite radiological extension being more prominent in male patients, quadricep muscle weakness was significantly greater among the female patients than in the male bronchiectasis patients—both absolute and predicted values. These observations are very relevant and as far as we are concerned these are novel findings that put the line forward that the lower limb muscles of female bronchiectasis patients are more severely affected than those of the males. These results also suggest that quadriceps muscle weakness was independent of the degree of the bronchiectasis radiological extension.

Moreover, from the study results it is also possible to conclude that the upper and lower limb muscles are not equally affected in bronchiectasis patients, the latter being more negatively altered, particularly in the female patients. These findings warrant further attention and future research should aim to identify the pathophysiology of the muscle abnormalities and gender differences within the quadriceps of bronchiectasis patients. Besides, the implications of lower limb muscle weakness towards exercise capacity should also be explored in future investigations. Moreover, similarly to what happens in other chronic lung diseases, namely COPD and in chronic heart failure patients, pulmonary rehabilitation and particularly exercise training are essential therapeutic strategies to improving muscle function, structure, and performance along with exercise tolerance and quality of life [10,44,56,57,58,59,60,61,62,63,64,65,66,67,68,69]. Hence, it is possible to conclude that pulmonary rehabilitation should be part of the wide spectrum of therapeutic tools currently available in patients with bronchiectasis. As such, pulmonary rehabilitation may be a component of the multidisciplinary therapeutic approach to be applied to specific phenotypes of patients. Future research should be devoted to this specific aspect in the management of patients with bronchiectasis.

In patients with bronchiectasis, inspiratory muscle function, as measured by MIP absolute and predicted values, was reduced, and such a decrease was observed particularly in the female patients compared to the female controls. Furthermore, a significant decline was also observed in SNIP and MEP absolute and predicted values in the patients as a whole and in both male and female patients, as compared to their respective controls. Taken together, these findings reveal that the function of inspiratory and expiratory muscles is significantly altered in bronchiectasis patients. These findings are consistent with previous results, in which a small cohort of bronchiectasis patients also demonstrated a significant decline in MIP and MEP absolute and predicted values compared to healthy controls [70]. The novelty in our study relies on the reported gender differences and on the use of SNIP as a reliable measurement of inspiratory muscle function [44,71]. Importantly, negative associations were also found between either disease severity scores or radiological extension and MIP and MEP parameters, suggesting that patients with greater score values were those with lower respiratory muscle performance.

## 5. Study Critique

Reference values used in the present study were those published in the literature, in which the phenotypic features of the participants were similar to those of our patients. Moreover, precise reference had to be used for each specific type of the measurements performed in this study. Thus, reference values have been customized to each particular measurement and similarities of the phenotypic characteristics.

Whether the assessment of patients with other concomitant respiratory diseases, such as asthma or COPD, may have yielded similar results should be a matter of research in future investigations. In fact, bronchiectasis has been recently proposed to be one of the most relevant asthma-associated comorbidities [72,73], and the combination of these diseases may worsen clinical outcomes, including muscle dysfunction.

Another potential limitation is related to the lack of use of specific questionnaires to assess physical activity or detailed diet components in the patients and healthy controls. Nonetheless, the participants were all inquired whether they were conducting any outdoor or indoor regular high-intensity physical activity or following any specific exercise training program.

## 6. Conclusions

In patients with mild-to-moderate bronchiectasis, respiratory and peripheral muscle function is significantly impaired and only partly related to the status of lung disease. Quadricep muscle strength was particularly weakened in female patients and was negatively associated with their exercise tolerance but not with the extent of the bronchiectasis. The results reported herein have clinical implications in the clinical management of these patients. Specific therapeutic strategies targeted to improving muscle mass and performance should be applied to bronchiectasis patients with peripheral muscle weakness. Body composition and peripheral muscle function determination should be part of the comprehensive clinical assessment of these patients.

## Figures and Tables

**Table 1:** Clinical characteristics and functional status of bronchiectasis patients and healthy controls.

	Healthy Controls	Bronchiectasis Patients
	N = 37	N = 150
Age, years, $\bar{x}$ (SD)	62.4 (9.8)	64.6 (13.2)
**Disease severity,**$\bar{x}$ (SD)		
FACED score	NA	1.67 (1.40)
EFACED score	NA	1.94 (1.64)
BSI score	NA	5.74 (3.42)
Exacerbations in previous year	NA	0.91 (1.11)
Hospitalizations for exacerbations	NA	0.21 (0.66)
Chronic colonization by PA, N (%)	NA	19 (12.7)
**Radiological extension**, $\bar{x}$ (SD)		
Total extent bronchiectasis score	NA	7.6 (3.6)
Bronchial dilatation score	NA	1.2 (0.3)
Bronchial wall thickness score	NA	1.2 (0.3)
Global severity score	NA	10 (3.5)
**Smoking history**		
Current smokers, N (%)	0	7 (5)
Ex-smokers, N (%)	0	51 (34)
Never smokers, N (%)	37	92 (61)
Packs-year, $\bar{x}$ (SD)	0	20.5 (16.7)
**Lung function,**$\bar{x}$ (SD)		
FEV_1_, % predicted	99 (12)	74 (22) ***
FVC, % predicted	100 (12)	83 (20) ***
FEV_1_/FVC, %	78 (6)	68 (13) ***
DL_CO_, % predicted	92 (6)	75 (16) ***
K_CO_, % predicted	88 (13)	78 (14) *
RV, % predicted	107 (8)	147 (35) ***
TLC, % predicted	97 (7)	101 (16)
RV/TLC, %	38 (3)	54 (10) ***
**Exercise capacity,**$\bar{x}$ (SD)		
6-min walking distance, meters	533 (68)	463 (99) ***
Distance, % predicted	106 (13)	95 (19) ***
Initial oxygen saturation, %	98 (1)	96 (2) ***
Medium oxygen saturation, %	97 (2)	94 (3) ***
Minimum oxygen saturation, %	96 (2)	93 (4) ***
Final oxygen saturation, %	96 (2)	93 (4) ***
**Blood parameters,**$\bar{x}$ (SD)		
CRP, mg/dL	0.2 (0.1)	0.6 (0.9) ***
ESR, mm/h	5.7 (3.9)	11.0 (9.8) **
Fibrinogen, mg/dL	333.6 (63.7)	393.8 (97.2) ***
Alpha-1 antitrypsin	118.9 (16.9)	135.3 (27.2) **
Hemoglobin, g/dL	14.4 (1.3)	13.9 (1.9) *
Hematocrit, %	43.1 (3.8)	42.2 (4.8)
Creatinine, mg/dL	0.8 (0.2)	0.8 (0.2)
Total proteins, g/dL	7.2 (0.3)	7.1 (0.5)
Albumin, g/dL	4.6 (0.2)	4.4 (0.3) ***
Prealbumin, g/dL	26.3 (4.5)	22.7 (5.2) **

Continuous variables are presented as mean (standard deviation), while categorical variables are presented as the number of patients in each group along with the percentage for the study group. Definition of abbreviations: $\bar{x}$, mean; SD, standard deviation; N, number; NA, not applicable; FACED: F, FEV_1;_ A, age; C, chronic colonization by *Pseudomonas aeruginosa* (PA); E, radiologic extension; D, dyspnea; BSI, bronchiectasis severity index; FEV_1_, forced expiratory volume in the first second; FVC, forced vital capacity; RV, residual volume; TLC, total lung capacity; DLco, carbon monoxide transfer; K_CO_, Krogh transfer factor; CRP, C-reactive protein; ESR, erythrocyte sedimentation rate; g, grams; dL, deciliter; mg, milligrams; mm, millimeters; h, hour. Statistical analyses and significance: * *p* < 0.05; ** *p* < 0.01; *** *p* < 0.001 between bronchiectasis patients and healthy controls.

**Table 2:** Clinical characteristics and functional status in bronchiectasis patients and healthy controls according to gender differences.

	Healthy Controls		Bronchiectasis Patients	
	Women	Men	Women	Men
	N = 21	N = 16	N = 114	N = 36
Age, years	63.4 (9.7)	61 (10.1)	65.4 (12.4)	62.0 (15.3)
**Disease severity**				
BSI	NA	NA	5.98 (3.57)	4.97 (2.80) §
EFACED	NA	NA	2.08 (1.75)	1.5 (1.16) §
FACED	NA	NA	1.76 (1.47)	1.39 (1.10)
Exacerbations in previous year	NA	NA	0.94 (1.17)	0.83 (0.88)
Hospitalization for exacerbations	NA	NA	0.25 (0.73)	0.08 (0.37)
Chronic colonization by PA	NA	NA	14 (12.3)	5 (13.9)
**Radiological extension,**$\bar{x}$ (SD)				
Total extent of bronchiectasis score	NA	NA	7.3 (3.5)	8.7 (3.5) §
Bronchial dilatation score	NA	NA	1.2 (0.4)	1.1 (0.2)
Bronchial wall thickness score	NA	NA	1.2 (0.3)	1.2 (0.2)
Global severity score	NA	NA	9.7 (3.5)	11 (3.6) §
**Smoking history**				
Current smokers, N	0	0	6 (5)	1 (3)
Ex-smokers, N	0	0	35 (31)	16 (44)
Never smokers, N	21 (100)	16 (100)	73 (64)	19 (53)
Packs-year,$\bar{x}$ (SD)	0	0	21.3 (15.2)	18.5 (20.6)
**Lung function,**$\bar{x}$ (SD)				
FEV_1_, % predicted	101 (12)	96 (13)	74 (22) ***	74 (23) **
FVC, % predicted	100 (12)	100 (12)	84 (20) **	83 (21) *
FEV_1_/FVC, %	80 (5)	75 (7)	67 (13) ***	68 (11)
DL_CO_, % predicted	88 (8)	94 (4)	74 (16) *	77 (16) *
K_CO_, % predicted	82 (3)	92 (15)	76 (15)	84 (11) *
RV, % predicted	107 (9)	106 (8)	152 (36) *	132 (28) *§
TLC, % predicted	98 (6)	97 (8)	103 (15)	93 (15) *
RV/TLC, %	39 (1)	38 (4)	57 (10) *	47 (7) ** §§§
**Exercise capacity,**$\bar{x}$ (SD)				
6-min walking distance, meters	496 (60)	579 (44)	447 (94) *	512 (99) * §§
Distance, % predicted	105 (10)	108 (15)	94 (18) *	97 (23) *
Initial oxygen saturation, %	98 (1)	98 (1)	96 (2) **	96 (2) **
Medium oxygen saturation, %	97 (2)	97 (1)	94 (3) **	94 (3) **
Minimum oxygen saturation, %	96 (2)	97 (2)	92 (4) ***	93 (3) **
Final oxygen saturation, %	96 (2)	97 (2)	92 (4) ***	93 (3) **
**Blood parameters,**$\bar{x}$ (SD)				
CRP, mg/dL	0.2 (0.1)	0.2 (0.1)	0.6 (0.9) *	0.6 (0.6) *
ESR, mm/h	6.6 (3.6)	4.7 (4.0)	11.3 (9.9) *	10 (9.6) *
Fibrinogen, mg/dL	349.5 (64)	313.8 (59.3)	397.4 (88.6) *	381.8 (122.1) *
Alpha-1 antitrypsin, mg/dL	117.5 (19.0)	121.2 (13.4)	134.3 (24.5) **	138.7 (35.0) *
Hemoglobin, g/dL	13.7 (1.0)	15.2 (1.0)	13.7 (2.1)	14.5 (1.0) * §
Hematocrit, %	41.3 (3.4)	45.6 (2.9)	41.7 (5)	43.9 (3.6) §
Creatinine, mg/dL	0.7 (0.1)	1.0 (0.1)	0.7 (0.1)	1.0 (0.2) §§§
Total proteins, g/dL	7.2 (0.3)	7.2 (0.3)	7.1 (0.4)	7.2 (0.5)
Albumin, g/dL	4.6 (0.2)	4.6 (0.2)	4.4 (0.3) **	4.4 (0.4) *
Prealbumin, g/dL	25.5 (4.7)	27.6 (3.9)	21.8 (4.7) **	25.3 (5.9) §§

Continuous variables are presented as mean (standard deviation), while categorical variables are presented as the number of patients in each group along with the percentage for the study group. Definition of abbreviations: $\bar{x}$, mean; SD, standard deviation; N, number; NA, not applicable; FACED: F, FEV_1;_ A, age; C, chronic colonization by *Pseudomonas aeruginosa* (PA); E, extension radiologic; D, dyspnea; BSI, bronchiectasis severity index; FEV_1_, forced expiratory volume in the first second; FVC, forced vital capacity; RV, residual volume; TLC, total lung capacity; DLco, carbon monoxide transfer; K_CO_, Krogh transfer factor; CRP, C-reactive protein; ESR, erythrocyte sedimentation rate; g, grams; dL, deciliter; mg, milligrams; mm, millimeters; h, hour. Statistical analyses and significance: * *p* ≤ 0.05; ** *p* ≤ 0.01; *** *p* ≤ 0.001 between healthy and bronchiectasis women or healthy and bronchiectasis men; § *p* ≤ 0.05; §§ *p* ≤ 0.01; §§§ *p* ≤ 0.001 between men and women patients.

## Data Availability

The datasets are available from the corresponding authors upon reasonable request.
